# The Multiplex qPCR Assay Enables Simultaneous Detection, Differentiation, and Quantification of Feline Bocavirus‐1, ‐2, and ‐3 in Clinical Samples

**DOI:** 10.1155/tbed/3455556

**Published:** 2026-07-01

**Authors:** Pattiya Lohavicharn, Tanit Kasantikul, Chutchai Piewbang, Somporn Techangamsuwan

**Affiliations:** ^1^ Department of Pathology, Faculty of Veterinary Science, Chulalongkorn University, Bangkok, 10330, Thailand, chula.ac.th; ^2^ Center of Excellence in Animal Virome and Diagnostic Development, Faculty of Veterinary Science, Chulalongkorn University, Bangkok, 10330, Thailand, chula.ac.th; ^3^ Veterinary Diagnostic Laboratory, Department of Pathobiology and Diagnostic Investigation, College of Veterinary Medicine, Michigan State University, East Lansing, Michigan, USA, msu.edu

**Keywords:** feline bocavirus, multiplex qPCR, simultaneous detection, species differentiation, viral load quantification

## Abstract

Feline bocaviruses (FBoVs) are increasingly recognized for their global distribution and potential importance in feline health. Three distinct species—FBoV‐1, FBoV‐2, and FBoV‐3—have been identified; however, the lack of efficient molecular assays has limited the ability to clarify their species distribution, prevalence, and viral loads. In this study, we developed a multiplex quantitative PCR (qPCR) assay targeting the *NS1* gene for the simultaneous detection, differentiation, and quantification of all three FBoV species. The assay demonstrated high analytical sensitivity, with limits of detection of 1.6 × 10^3^ copies/μL for FBoV‐1 and 1.6 × 10^2^ copies/μL for both FBoV‐2 and FBoV‐3. No cross‐reactivity was observed with other feline or non‐feline viruses, and the assay showed strong repeatability and reproducibility. Diagnostic evaluation using 135 archived feline oropharyngeal and fecal samples yielded a sensitivity of 95.2% and a specificity of 100% compared with previously established singleplex qPCR assays. Importantly, the multiplex format reduced time, cost, and labor while maintaining high diagnostic accuracy. This study presents the first multiplex qPCR assay capable of simultaneously detecting, differentiating, and quantifying all three FBoV species, providing a rapid, sensitive, and specific tool for routine diagnostics and large‐scale epidemiological investigations.

## 1. Introduction

Feline bocaviruses (FBoVs) are nonenveloped, single‐stranded DNA viruses of approximately 5.2−5.5 kb in length belonging to the genus *Bocaparvovirus*, family *Parvoviridae*. Their genomes contain three major open reading frames (ORFs) encoding the nonstructural protein NS1, the unique nonstructural protein NP1, and the structural capsid proteins VP1/2 [1]. The *NS1* gene is commonly used for bocavirus classification and species differentiation. The NS1 region is conserved within each bocavirus species but differs between species, making it a suitable target for species‐specific detection assays. In contrast, the *NP1* gene is exclusive to bocaviruses and is involved in post‐transcriptional regulation, making it a useful target for panbocavirus detection assays [1–4].

To date, three genetically distinct FBoV species have been identified: FBoV‐1 (species Bocaparvovirus carnivoran 3), FBoV‐2 (species Bocaparvovirus carnivoran 4), and FBoV‐3 (species Bocaparvovirus carnivoran 5) [4–6]. FBoV‐1 was first reported in Hong Kong in 2012 [4], followed by detections of FBoV‐2 and FBoV‐3 in the United States, Japan, China, Portugal, Turkey, Thailand, and other regions, indicating a global distribution and ongoing viral diversification [7–10]. Sequence analyses suggest continuous evolution and recombination among circulating strains [7, 11].

Epidemiological surveys have revealed variable prevalence among feline populations. For instance, in northeast China, FBoV was detected in 25.9% of fecal samples from 197 cats, with a higher rate in diarrheic cats (33.3%) than in healthy cats (17.4%) [3]. Similarly, FBoV DNA has been detected in feces, respiratory secretions, and blood of both healthy and clinically ill cats [4, 7]. Although FBoV‐1 has been associated with enteric diseases and hemorrhagic enteritis in some outbreaks [7, 11], the pathogenic roles of FBoV‐2 and FBoV‐3 remain uncertain. The frequent detection of coinfections with other enteric viruses—such as feline panleukopenia virus (FPLV), feline astrovirus, and feline coronavirus (FCoV)—further complicates interpretation [5, 6].

Early diagnostic efforts relied primarily on conventional PCR assays targeting *NP1* or *NS1* genes, which permitted FBoV screening but did not distinguish species without sequencing [8, 9]. To overcome these limitations, quantitative PCR (qPCR) platforms were developed [12–15]. These assays allowed viral load quantification, facilitating insights into infection dynamics. Although several qPCR assays have been developed [12–15], most target only FBoV‐1, leaving FBoV‐2 and FBoV‐3 insufficiently characterized. While a few strains of these species have been identified through metagenomic studies [8, 9], information on their prevalence, geographic distribution, and coinfection patterns remains limited.

Recognizing the need for simultaneous detection of all known species, subsequent studies developed singleplex qPCR assays targeting the *NS1* gene for FBoV‐1, FBoV‐2, and FBoV‐3 [7, 11]. The *NS1* region provides an optimal target because it is conserved within species but sufficiently diverse among them for differentiation. However, singleplex assays are labor‐intensive, increasing the cost and turnaround time in large‐scale testing.

In this study, we designed and validated a TaqMan‐based multiplex qPCR assay targeting the *NS1* gene for the simultaneous detection, species differentiation, and quantification of the three recognized FBoV species—FBoV‐1, FBoV‐2, and FBoV‐3. Analytical sensitivity, specificity, efficiency, reproducibility, and repeatability were comprehensively evaluated. The resulting assay provides a rapid, sensitive, and reliable tool for detecting all known FBoV species in a single reaction, enabling large‐scale epidemiological and clinical studies in feline populations.

## 2. Materials and Methods

### 2.1. Ethics Statement

All experimental protocols were approved by the Chulalongkorn University Animal Care and Use Committee (IACUC Number 2231029) and the Institutional Biosafety Committee (IBC Number 2231032) of Chulalongkorn University, Bangkok, Thailand. All procedures were conducted in accordance with the ARRIVE guidelines. Informed consent was obtained from animal owners prior to sample collection or carcass submission.

### 2.2. Standard Plasmid Control

The *NS1* regions covering the target areas of each FBoV species were trimmed and aligned using BioEdit version 7.2.5. The aligned sequences were analyzed, and representative *NS1* sequences reflecting the majority of circulating FBoV strains, particularly Thai isolates, were selected. The reference sequences included an unpublished Thai strain for FBoV‐1, PP592983 for FBoV‐2, and PP592989 for FBoV‐3.

A 500‐bp DNA fragment containing the selected *NS1* target region was synthesized and cloned into the pMARQ (AmpR) plasmids vector, followed by transformation into *E. coli* K12 DH10B T1R (Invitrogen, CA, USA), yielding a recombinant plasmid of approximately 2841 bp in total length. The DNA concentration was measured using a NanoDrop spectrophotometer (Thermo Scientific, MA, USA). Plasmid copy number was calculated using the following formula:

Copies/µL=6.022×1023×DNA concentration g/µLPlasmid length bp×660.

Plasmids were resuspended and diluted into 1.6 × 10^10^ copies/µL and stored at −80°C until further use.

### 2.3. Primer and Probe Design

All available complete coding sequences of FBoVs were retrieved from the GenBank NCBI database, then compiled, and aligned using BioEdit version 7.2.5 and MAFFT version 7, respectively [16]. Sequence alignments were manually inspected to identify regions suitable for multiplex qPCR assay development. The *NS1* gene was selected as the target region. Primer and probe sets for each assay were subsequently designed and evaluated using in silico analyses.

Primer and probe design followed established qPCR design principles [17]. Key design parameters included primer length (18−30 bp), GC content (35%−65%), and target amplicon length (70−150 bp). The melting temperature (Tm) difference among primers was maintained within 2°C, and probe Tm values were set approximately 5−10°C higher than those of the primers. Hairpin Tm was required to be lower than the primers Tm. Additionally, recommended limits for unfavorable Δ*G* values and 3′ end interactions were considered to ensure compatibility within the multiplex format.

In silico analysis was conducted to evaluate the specificity and secondary structures of the candidate primers and TaqMan probes. Each designed primer and probe sequence was assessed using the BLASTn program [18] to confirm species specificity. Given the high genomic similarity among FBoV species, cross‐checking against nontarget FBoVs was performed by specifying the target species in BLASTn. Secondary structure characteristics—including Tm, hairpin formation, self‐dimerization, and hetero‐dimerization—were assessed using OligoAnalyzer (https://sg.idtdna.com/calc/analyzer).

### 2.4. Clinical Sample Preparation and Nucleic Acid Extraction

A total of 135 archived samples, consisting of oropharyngeal or fecal swabs collected from domestic cats in Thailand between 2018 and 2022 for routine diagnostic investigations, were included. All samples had previously been tested for FBoV by in‐house diagnostic PCR and had been stored at −80°C [7, 19–21]. All FBoV‐positive samples were also confirmed by sequencing, representing three known FBoV species—FBoV‐1, FBoV‐2, and FBoV‐3.

To avoid selection bias, each archived sample was assigned a unique ID and randomly selected using an online random number generator [22]. Of these 135 samples, 73 were FBoV‐negative, and 62 were FBoV‐positive. The FBoV‐positive samples consisted of 45 FBoV‐1, three FBoV‐2, and six FBoV‐3 single‐infection samples. In addition, eight coinfection cases were identified, comprising seven FBoV‐1/FBoV‐2 coinfections and one FBoV‐2/FBoV‐3 coinfection.

Selected archived raw clinical specimens were thawed on ice prior to processing. Each swab sample, previously collected using sterile cotton swabs and resuspended in phosphate‐buffered saline (PBS) (pH 7.4), was vortexed briefly to ensure homogenization. The samples were then centrifuged briefly, and the supernatant was used for nucleic acid extraction. Viral DNA was extracted using the Viral Nucleic Acid Extraction II kit (Geneaid, Taipei, Taiwan) according to the manufacturer’s instructions. The extracted nucleic acids were stored at −80°C until further analysis.

In addition, viral nucleic acids obtained from vaccines containing FPLV, feline calicivirus (FCV), feline herpesvirus 1 (FHV‐1), and feline leukemia virus (FeLV) (Nobivac Feline 1‐HCP and Nobivac FeLV; Merck & Co., Inc., NJ, USA), as well as nucleic acids extracted from sequencing‐confirmed archived samples positive for FCoV, feline immunodeficiency virus (FIV), canine bocavirus 2 (CBoV‐2), and human bocavirus 1 (HBoV‐1) [23, 24], were included for assay validation.

### 2.5. Singleplex and Multiplex qPCR Optimization

Singleplex qPCR assays for FBoV‐1, FBoV‐2, and FBoV‐3 were previously established [20]. Briefly, reactions were performed in a 20 µL total volume containing 400 nM of each primer, 200 nM of TaqMan probe, and 2 µL of template DNA using 10 µL qPCRBIO Probe Mix Lo−ROX (PCR Biosystems Ltd., London, UK). The cycling conditions were as follows: 95°C for 3 min, followed by 40 cycles of 95°C for 10 s and 60°C for 20 s.

For the multiplex qPCR assay, optimization was performed based on the singleplex qPCR assay protocol using the same reagents and cycling conditions described above. Primer and probe concentrations ranging from 100–400 nM and annealing temperatures (*T*_*a*_) ranging from 54–62°C were tested using standard positive controls. The combination of primer–probe concentrations and cycling conditions that yielded the lowest cycle threshold (*C*_*t*_) value and consistent amplification efficiency was selected for the final assay configuration. All reactions were conducted on a Rotor‐Gene Q real‐time PCR system (Qiagen, Hilden, Germany).

### 2.6. Analytical Sensitivity and Spiking Test of the Multiplex qPCR Assay

Standard plasmids were serially diluted 10‐fold from the stock solution to achieve concentrations ranging from 10^9^ to 10^1^ copies/µL. These serial dilutions were used to generate standard curves and served as templates for evaluating the analytical sensitivity of the assay. Each experiment was performed under optimized conditions, and the analytical sensitivity, defined as the limit of detection (LOD), was determined accordingly.

To further validate the reliability of the LOD of the developed multiplex qPCR under biological sample conditions, a spiking test was performed [24] by adding known concentrations of the positive plasmid standard into the FBoV‐negative fecal samples. Briefly, 198 µL of the fecal sample was aliquoted and spiked with 2 µL of plasmid standard at the LOD concentration, as well as 1–2 concentrations above the LOD. Then, the nucleic acid was extracted using the Viral Nucleic Acid Extraction II kit (Geneaid, Taipei, Taiwan) according to the manufacturer’s protocols.

### 2.7. Analytical Specificity of the Multiplex qPCR Assay

To assess the analytical specificity of the assays, cross‐reactivity tests were performed against several common feline viruses, including FPLV, FCoV, FCV, FHV‐1, FeLV, and FIV. In addition, other bocavirus species, including CBoV‐2 and HBoV‐1, were tested. To further confirm species‐level specificity, cross‐reactivity among the three FBoV species examined by testing each primer−probe set against the plasmid controls of the other two species. Samples with a Ct value greater than 35 were considered negative.

### 2.8. Repeatability and Reproducibility of the Multiplex qPCR Assay

The repeatability (intra‐assay variation) and reproducibility (inter‐assay variation) of the assay were evaluated using plasmid standards for FBoV‐1, FBoV‐2, and FBoV‐3. Plasmid concentrations were tested under optimized assay conditions. To verify the assay reliability, all samples were analyzed in triplicate across three independent runs. Results were reported as the mean quantification cycle (*C*_*q*_) value, standard deviation (SD), and coefficient of variation (CV).

### 2.9. Comparative Evaluation of Singleplex and Multiplex qPCR Assays

The performance of the optimized multiplex qPCR assay was evaluated by comparison with established singleplex qPCR assays [20]. To access diagnostic sensitivity (DSe) and diagnostic specificity (DSp), a total of 135 archived fecal samples were analyzed, comprising 62 FBoV‐positive and 73 FBoV‐negative samples. Positive samples included FBoV‐1, FBoV‐2, and FBoV‐3 which were previously tested by PCR [4] and confirmed by sequencing. Diagnostic performance metrics were calculated as follows: DSe, DSp, positive predictive value (PPV), negative predictive value (NPV), positive likelihood ratio (LR^+^), and negative likelihood ratio (LR^−^). Each estimate was reported with a 95% confidence interval (CI) using the Clopper–Pearson (binomial exact) method. The agreement between the multiplex and singleplex qPCR assays was evaluated using the chi‐square test. All statistical analyses were performed in *R* software (version 4.3.1; R Core Team, Vienna, Austria) using the epiR package (version 2.0.66). A *p*‐value <0.05 was considered statistically significant.

## 3. Results

### 3.1. Primer and Probe Pairs

Following sequence alignment and manual inspection of conserved and variable regions within the *NS1* gene, several candidate primer and probe sites were identified for FBoV‐1, FBoV‐2, and FBoV‐3 based on qPCR‐designed criteria. Subsequently, in silico analyses were used to select the optimal primer–probe sets for all three FBoV assays to be combined into a single multiplex qPCR assay.

The selected primers and probes were 19–24 nucleotides in length, with closely matched melting temperatures for primers (49.5–51.4°C) and probes (58–61.1°C). All predicted amplicon sizes were within the optimal range, which was 149 bp for FBoV‐1, 110 bp for FBoV‐2, and 126 bp for FBoV‐3. All primers and probes revealed high predicted specificity in BLASTn searches, with no significant matches to nontarget FBoVs and other feline common viruses or the feline host genome. Secondary structure analyses also indicated that all primers and probes were structurally suitable for multiplexing. The self‐dimer Δ*G* values ranged from −1.61 to −5.36 kcal/mol, and the predicted hairpin structures showed Δ*G* values between −40.6 and 40.2 kcal/mol. Most hetero‐dimer interactions were also minimal, with Δ*G* values above −9.0 kcal/mol, except for a few pairs that showed slightly lower values (−9.16, −9.8, and −10.22 kcal/mol), which still fell within tolerable ranges for multiplex qPCR.

To establish the multiplex qPCR assay, a distinct fluorophore was assigned to each FBoV probe. Each of the fluorophores was assigned as fluorescein amidite (FAM) for FBoV‐1, hexachlorofluorescein (HEX) for FBoV‐2, and cyanine 5 (Cy5) for FBoV‐3 according to the probe position and the amplicon size. All primer and probe sequences are listed in Table 1, and their target regions are illustrated in Figures S1−S3.

**Table 1:** Primers and probes targeting the *NS1* region for the multiplex qPCR assay.

Virus	Name	Sequence 5′–3′	Target (bp)
FBoV‐1	FBoV1_1127F	AAAGATTGTTCCGTATCACG	149
	FBoV1_1276R	CCCATAGTACCAGTTAATTTCC	
	Probe	FAM‐AAGATCTCCAAACACGTCTCCCTT‐BHQ1	

FBoV‐2	FBoV2_864F	GCACTCTCGTTAATAAACAAC	110
	FBoV2_974R	GTAAAGTCCACCTCCTCTA	
	Probe	HEX‐CTAAACGACACTGCCAACCTGG‐BHQ1	

FBoV‐3	FBoV3_1194F	GAAGAACCTATATTCAAAGGAGAC	126
	FBoV3_1320R	TTTTGCTCATTCTAGCGTTTAC	
	Probe	CY5‐TGAGCACCACCGAGACCCAT‐BHQ2	

Abbreviations: BHQ1, Black Hole Quencher 1; BHQ2, Black Hole Quencher 2; CY5, cyanine 5; F, forward primer; FAM, fluorescein amidite; HEX, hexachlorofluorescein; R, reverse primer.

### 3.2. Optimization of Multiplex qPCR

The multiplex qPCR assay was initially developed under singleplex qPCR conditions using Ta at 60°C, with primer and probe concentrations of 400 and 200 nM, respectively. Under these conditions, nonspecific amplicons and early false‐positive dimer signals (Ct 20–25) were observed and confirmed by a capillary electrophoresis device (QIAxcel DNA Screening Kit; Qiagen). Late amplification signals were occasionally observed in the FBoV‐1 fluorescence channel, particularly in no‐template controls (NTCs) and highly diluted plasmid standards. Capillary electrophoresis demonstrated only low‐molecular‐weight bands consistent with primer–dimer formation rather than the expected target amplicon in these reactions (Figure S4A).

To reduce the dimer formation, primer and probe concentrations were lowered to 200 and 150 nM, respectively, while maintaining the Ta at 60°C. However, nonspecific primer–dimer signals were still observed at Ct values > 35. A range of Ta (54, 58, and 62°C) was subsequently tested to improve assay specificity and minimize dimer formation, but these conditions reduced the overall assay efficiency compared with the 60°C condition.

**Figure 1:**
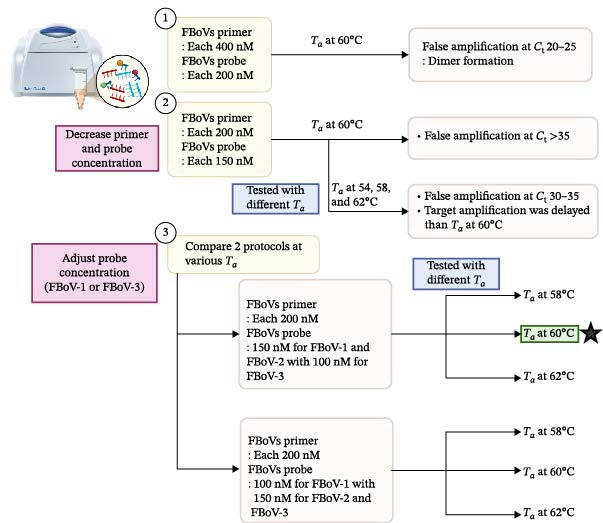
Optimization workflow for multiplex qPCR assay development. Three rounds of optimization were performed to eliminate nonspecific amplification and improve assay performance. Different combinations of primer and probe concentrations, as well as various annealing temperatures (T_a_), were systematically evaluated. The results of each round were used to guide subsequent adjustments. The final optimized conditions were T_a_ of 60°C, with primer concentrations of 200 nM for all targets and probe concentrations of 150 nM for FBoV‐1 and FBoV‐2, and 100 nM for FBoV‐3, indicated by the star (★).

Because the dimer patterns suggested potential interactions involving the FBoV‐1 and FBoV‐3 primer–probe sets (Figure S4B), different probe concentrations for FBoV‐1 and FBoV‐3 were further optimized as a result. Two conditions were compared: (i) 200 nM primer and 150 nM probe for FBoV‐1 and FBoV‐2, and 100 nM probe for FBoV‐3; and (ii) 200 nM primer and 100 nM probe for FBoV‐1, and 150 nM probe for FBoV‐2 and FBoV‐3. Both conditions were tested at Ta of 58, 60, and 62°C. Based on these evaluations, the optimal multiplex qPCR conditions were Ta of 60°C, with final primer concentrations of 200 nM for all assays and probe concentrations of 150 nM for FBoV‐1 and FBoV‐2, and 100 nM for FBoV‐3. The optimization workflow is shown in Figure 1.

### 3.3. Standard Curve and Analytical Sensitivity

**Figure 2:**
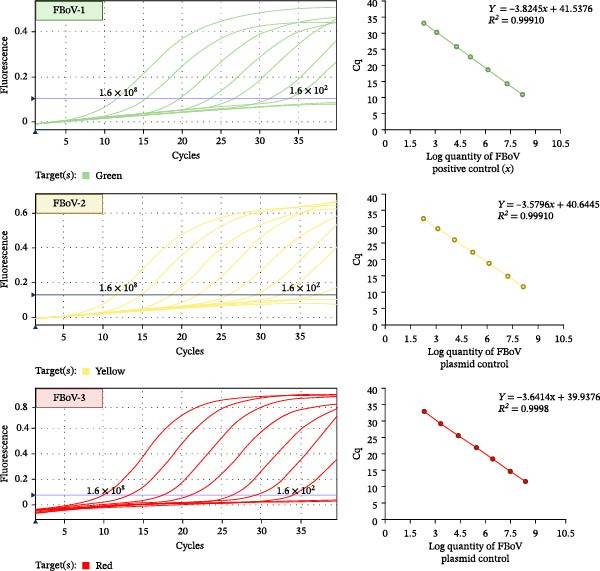
Analytical sensitivity of the multiplex qPCR assay for FBoV‐1, FBoV‐2, and FBoV‐3. Standard curves, limits of detection (LOD), and amplification plots for each FBoV species are shown. Amplification curves were generated from 10‐fold serial dilutions of plasmid standards ranging from 1.6 × 10^8^ to 1.6 × 10^0^ copies/µL. The highest dilution (10^8^) and the lowest reliably detected (10^2^) dilution are indicated. Linear regression analysis produced slopes between −3.57 and −3.82 with coefficients of determination (*R* ^2^) > 0.99, demonstrating excellent linearity and amplification efficiency. The LOD for all targets was 1.6 × 10^2^ copies/μL, with consistent amplification observed across replicates.

Standard curves for all FBoV species were generated using 10‐fold serial dilutions of the standard plasmid, with each tested in triplicate. The assays produced slopes ranging from −3.57 to −3.82 and strong coefficients of determination (*R* ^2^ > 0.99), indicating high amplification efficiency and excellent linearity. The amplification efficiencies were 82.6%, 90.3%, and 88.2% for FBoV‐1, FBoV‐2, and FBoV‐3, respectively. The LOD for all three FBoV species was 1.6 × 10^2^ copies/μL, with consistent and reliable amplification observed up to cycle 35. Representative standard curves and amplification plots are shown in Figure 2.

### 3.4. Analytical Specificity and Spiking Test

**Figure 3:**
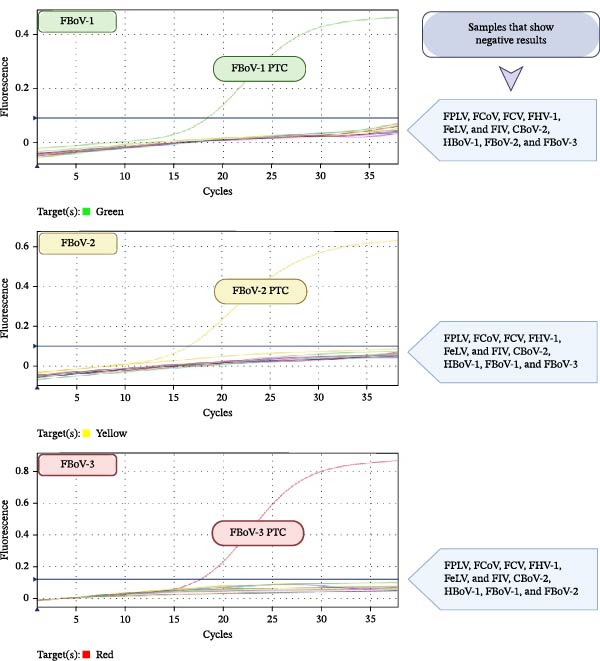
Specificity test of the multiplex qPCR assay for FBoV‐1, FBoV‐2, and FBoV‐3. Amplification curves are shown for the positive template controls (PTCs) of each target (green: FBoV‐1; yellow: FBoV‐2; red: FBoV‐3). No cross‐reactivity was detected with other feline pathogens (FPLV, FCoV, FCV, FHV‐1, FeLV, and FIV) or with nontarget bocaviruses (CBoV‐2, HBoV‐1, FBoV‐1, FBoV‐2, FBoV‐3).

No cross‐reactivity was observed among FBoV species or with other common feline viruses. In addition, no amplification was detected with CBoV‐2 or HBoV‐1. The results are presented in Figure 3.

**Figure 4:**
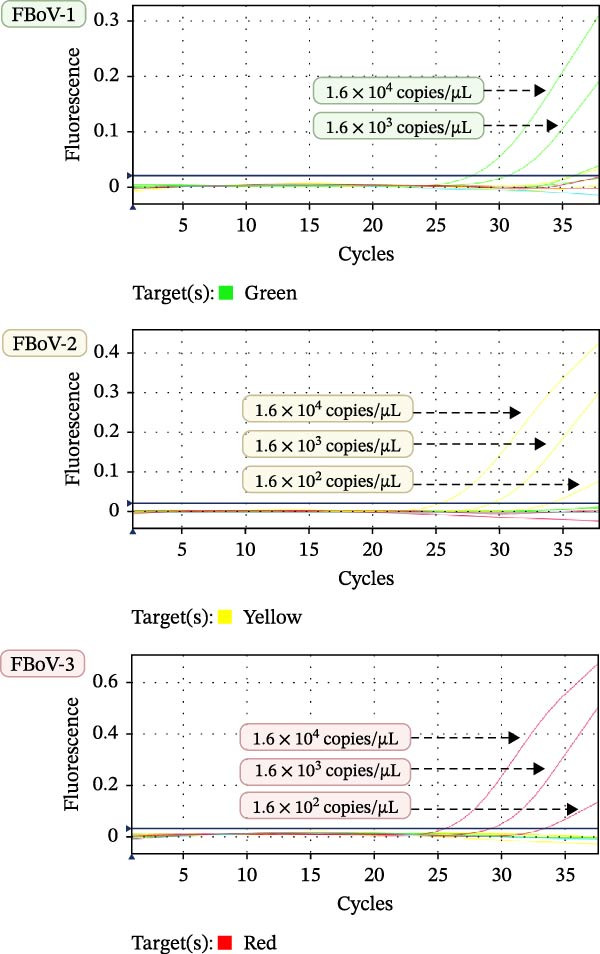
Spiking LOD analysis of the multiplex qPCR assay for FBoV‐1, FBoV‐2, and FBoV‐3. Three plasmid concentrations (1.6 × 10^2^, 1.6 × 10^3^, and 1.6 × 10^4^ copies/µL) were spiked into FBoV‐negative fecal samples. Consistent amplification was observed for FBoV‐2 and FBoV‐3 at 1.6 × 10^2^ copies/µL, while FBoV‐1 achieved at a higher concentration (1.6 × 10^3^ copies/µL). Colored labels and arrows highlight each concentration and correspond to the fluorescence signals of FBoV‐1 (green), FBoV‐2 (yellow), and FBoV‐3 (red).

The spiking LOD for FBoV‐2 and FBoV‐3 remained at 1.6 × 10^2^ copies/µL. In contrast, FBoV‐1 was not consistently detected at this concentration but showed reliable amplification at a higher dilution. Therefore, the spiking LOD for FBoV‐1 was adjusted to 1.6 × 10^3^ copies/µL. The results are presented in Figure 4.

### 3.5. Reproducibility and Repeatibility

All three FBoV assays were independently performed in three separate runs, with each run containing triplicate reactions of three selected plasmid dilutions (1.6 × 10^3^, 1.6 × 10^5^, and 1.6 × 10^7^ copies/µL), chosen based on the assay’s LOD. All assays showed high reproducibility and repeatability. The intra‐assay mean CVs ranged from 0.5% to 2.04% for FBoV‐1, 0.5%–1.23% for FBoV‐2, and 0.5%–1.44% for FBoV‐3, while the inter‐assay mean CVs remained between 0.29%–0.9%, 0.49–1.45%, and 0.97–1.9% for FBoV‐1, FBoV‐2 and FBoV‐3 respectively, as shown in Table 2.

**Table 2:** Repeatability (intra‐assay) and reproducibility (inter‐assay) of the multiplex qPCR assay for FBoV‐1, FBoV‐2, and FBoV‐3.

Virus	Dilution (sample size)	Input concentration (copies/µL)	Mean Cq ± SD	Intra‐assay mean CV (%)	Inter‐assay mean CV (%)
FBoV‐1	D‐3 (3)	1.6 × 10^7^	13.29 ± 0.24	2.04	0.44
	D‐5 (3)	1.6 × 10^5^	21.07 ± 0.19	0.5	0.9
	D‐7 (3)	1.6 × 10^3^	29.78 ± 0.28	0.93	0.29

FBoV‐2	D‐3 (3)	1.6 × 10^7^	12.38 ± 0.16	0.8	0.49
	D‐5 (3)	1.6 × 10^5^	20.19 ± 0.27	0.5	1.45
	D‐7 (3)	1.6 × 10^3^	28.53 ± 0.39	1.23	1

FBoV‐3	D‐3 (3)	1.6 × 10^7^	13.29 ± 0.24	1.44	1.9
	D‐5 (3)	1.6 × 10^5^	21.07 ± 0.19	0.5	1.26
	D‐7 (3)	1.6 × 10^3^	29.78 ± 0.28	0.89	0.97

Abbreviation: CV, coefficient value.

### 3.6. Comparative Evaluation of Singleplex and Multiplex qPCR Assays on Clinical Samples

The multiplex qPCR assays identified 59 of 62 positive samples (95.2%) and all 73 negative samples when compared with the singleplex qPCR assays (Table 3). The multiplex qPCR assay showed a DSe of 95.2% (95% CI: 86.5–99.0), specificity of 100% (95% CI: 95.1–100), PPV of 100% (95% CI: 93.9–100), NPV of 96.1% (95% CI: 88.9–99.2), LR^+^ = ∞, and LR^−^ = 0.05. Statistical analysis using the chi‐square test revealed no significant difference between the multiplex and singleplex assays (*p* > 0.05), confirming that both methods exhibit comparable diagnostic accuracy.

**Table 3:** Comparative diagnostic performance of the multiplex qPCR assay and singleplex qPCR assays using archived clinical fecal samples.

Sample classification by singleplex qPCR	Number of samples	Multiplex qPCR positive	Multiplex qPCR negative
FBoV‐positive samples	62	59	3
FBoV‐negative samples	73	0	73
Total	135	59	76

## 4. Discussion

Although research on FBoVs has expanded in recent years, their epidemiology remains poorly understood. One major limitation is the lack of diagnostic tools specifically designed for FBoV‐2 and FBoV‐3. Most previous studies relied on pan‐FBoV primers [4], which provide broad detection but require sequencing for species confirmation. Although several qPCR assays have recently been developed, most target only FBoV‐1 [12–15], leaving FBoV‐2 and FBoV‐3 insufficiently characterized. A few strains of these species have been identified through metagenomic approaches [8, 9]; however, information on their prevalence, geographic distribution, and coinfection patterns is still limited.

To address this knowledge gap, we expanded upon our previously developed singleplex qPCR assays for FBoV‐1, FBoV‐2, and FBoV‐3 [20] and successfully established a multiplex qPCR assay. Performing separate singleplex assays substantially increases the workload, reagent use, and cost in large‐scale surveillance; thus, a multiplex format offers a more efficient diagnostic alternative. Beyond reducing labor and resources, this multiplex assay enables simultaneous species differentiation and quantification of the three FBoV species in a single reaction, making it highly suitable for epidemiological and molecular studies. The assay was designed using primers targeting the *NS1* gene, a region that is highly conserved within each species yet sufficiently divergent among species to support accurate discrimination. Moreover, *NS1* is widely used for bocavirus classification [1], reinforcing its suitability for multiplex primer–probe design.

Validation results demonstrated that the assay is both highly sensitive and specific. Strong analytical sensitivity and diagnostic performance were achieved, with results consistent with the previously established singleplex assays. No cross‐reactivity was observed among FBoV species or with other common feline viruses or non‐FBoVs, including CBoV and HBoV, confirming that each primer–probe set was species‐specific and functioned reliably within the multiplex format.

To our knowledge, this is the first molecular tool capable of simultaneously detecting all three known FBoV species. In addition to rapid detection, the quantitative capability of the assay provides valuable opportunities for diverse study designs—including longitudinal, prospective, and cross‐sectional studies—to investigate viral load dynamics, shedding patterns, and persistence in infected animals. These data are essential for understanding potential associations between FBoV infection and disease outcomes.

Although the DSe of the multiplex assay did not differ significantly from that of the singleplex assays, its analytical sensitivity was slightly lower, reflecting a higher LOD. This modest reduction is an inherent constraint of multiplex platforms, where multiple primers and probes may compete for reaction components or form dimers, reducing the amplification efficiency [25]. Such effects may be more noticeable when simultaneously detecting genetically related targets, such as FBoV‐1, FBoV‐2, and FBoV‐3. Spiking experiments confirmed comparable detection limits for FBoV‐2 and FBoV‐3, whereas FBoV‐1 required a slightly higher template concentration for consistent amplification, which may reflect differences in primer efficiency or the presence of PCR inhibitors in clinical matrices. Nonetheless, the overall assay performance remained robust, demonstrating the practical benefits of multiplexing despite a small trade‐off in sensitivity.

In summary, this study presents a sensitive, specific, and quantitative multiplex qPCR assay that substantially enhance the ability to detect and characterize FBoVs. It provides an efficient tool for investigating FBoV prevalence, distribution, viral load, and coinfection patterns, forming a strong foundation for future epidemiological and comparative studies. Broader application of this assay may also increase awareness of FBoV infections and their potential impact on feline health. Future validation using larger and more diverse clinical and necropsy sample sets will be important to further confirm the assay performance under various field conditions. Integration of this multiplex qPCR method into ongoing feline virome investigations will strengthen surveillance efforts and contribute to a deeper understanding of bocavirus diversity, evolution, and possible cross‐species transmission.

## 5. Conclusion

A TaqMan‐based multiplex qPCR assay was successfully developed for the simultaneous detection, species differentiation, and quantification of FBoV‐1, FBoV‐2, and FBoV‐3. The assay demonstrated high sensitivity, specificity, and reproducibility, providing a reliable and efficient tool for both diagnostic screening and epidemiological studies of FBoVs.

## Funding

This research project is supported by the National Research Council of Thailand (NRCT) (Grant N41A640189), the 90^th^ Anniversary of Chulalongkorn University Fund (Ratchadaphiseksomphot Endowment Fund (to Pattiya Lohavicharn), the Second Century Fund (C2F) at the Chulalongkorn University (to Chutchai Piewbang and Pattiya Lohavicharn), and The Thailand Science Research and Innovation Fund, Chulalongkorn University (Grant HEA_FF_69_052_3100_004) (to Somporn Techangamsuwan).

## Conflicts of Interest

The authors declare no conflicts of interest.

## Supporting Information

Additional supporting information can be found online in the Supporting Information section.

## Supporting information


**Supporting Information** Alignment of the *NS1* gene sequences targeted by the multiplex qPCR assay for FBoV‐1, FBoV‐2, and FBoV‐3 are presented in Supporting Figure S1–S3, respectively. Representative capillary electrophoresis analysis during multiplex qPCR optimization is presented in Supporting Figure S4.

## Data Availability

All data supporting the findings of this study are available within the paper and its Supporting Information.
