# Supplementary material for: The Multiplex qPCR Assay Enables Simultaneous Detection, Differentiation, and Quantification of Feline Bocavirus‐1, ‐2, and ‐3 in Clinical Samples
Source: Transbound Emerg Dis. 2026 Jul 1;2026:3455556. doi: 10.1155/tbed/3455556 (PMC13319895; doi:10.1155/tbed/3455556)
Supplement: Supplementary file 1 — Supporting Information Alignment of the NS1 gene sequences targeted by the multiplex qPCR assay for FBoV‐1, FBoV‐2, and FBoV‐3 are presented in Supporting Figure S1–S3, respectively. Representative capillary electrophoresis analysis during multiplex qPCR optimization is presented in Supporting Figure S4. [file TBED-2026-3455556-s001.docx]

**Multiplex qPCR enables simultaneous detection, species differentiation, and quantification of feline bocavirus-1, -2, and -3 in clinical samples**

**Pattiya Lohavicharn^1,2^, Tanit Kasantikul^3^, Chutchai Piewbang^1,2^, Somporn Techangamsuwan^1,2,*^**

^1^Department of Pathology, Faculty of Veterinary Science, Chulalongkorn University, Bangkok, 10330, Thailand

^2^Center of Excellence in Animal Virome and Diagnostic Development, Faculty of Veterinary Science, Chulalongkorn University, Bangkok 10330, Thailand

^3^Veterinary Diagnostic Laboratory, Department of Pathobiology and Diagnostic Investigation, College of Veterinary Medicine, Michigan State University, East Lansing, MI, USA.

**
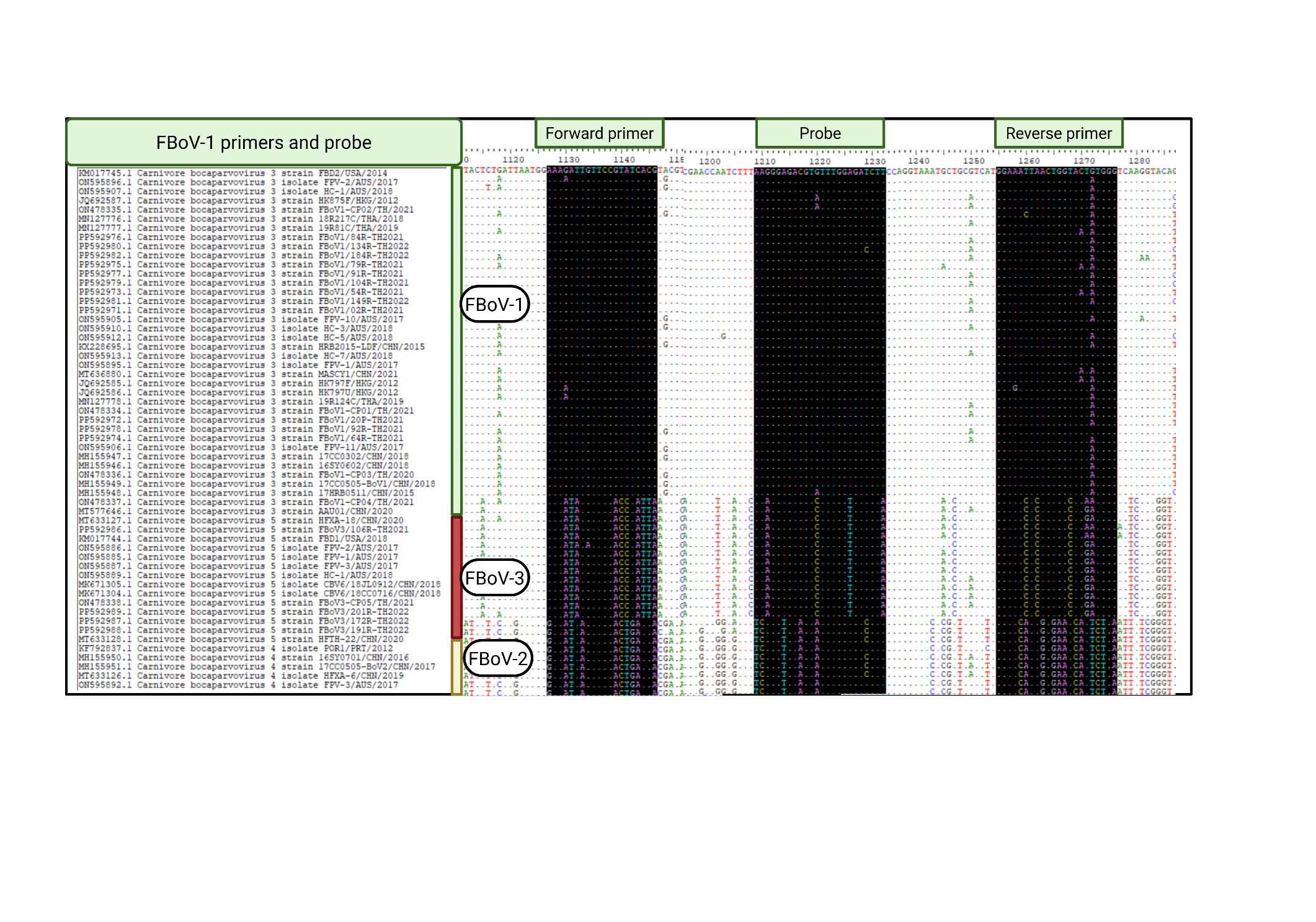
**

**Supplementary Figure S1. Alignment of the *NS1* gene sequences targeted by the multiplex qPCR assay for FBoV**-**1.** Multiple sequence alignment of the *NS1* gene region targeted by the FBoV**-**1–specific qPCR assay. All FBoV-1 sequences at the target region were compared with representative sequences of FBoV**-**2 and FBoV**-**3. The regions targeted by the forward primer, probe, and reverse primer are highlighted in black and enclosed within green rectangular boxes. The color bar on the left distinguishes the three bocaviruses: green for FBoV**-**1, yellow for FBoV**-**2, and red for FBoV**-**3.

**
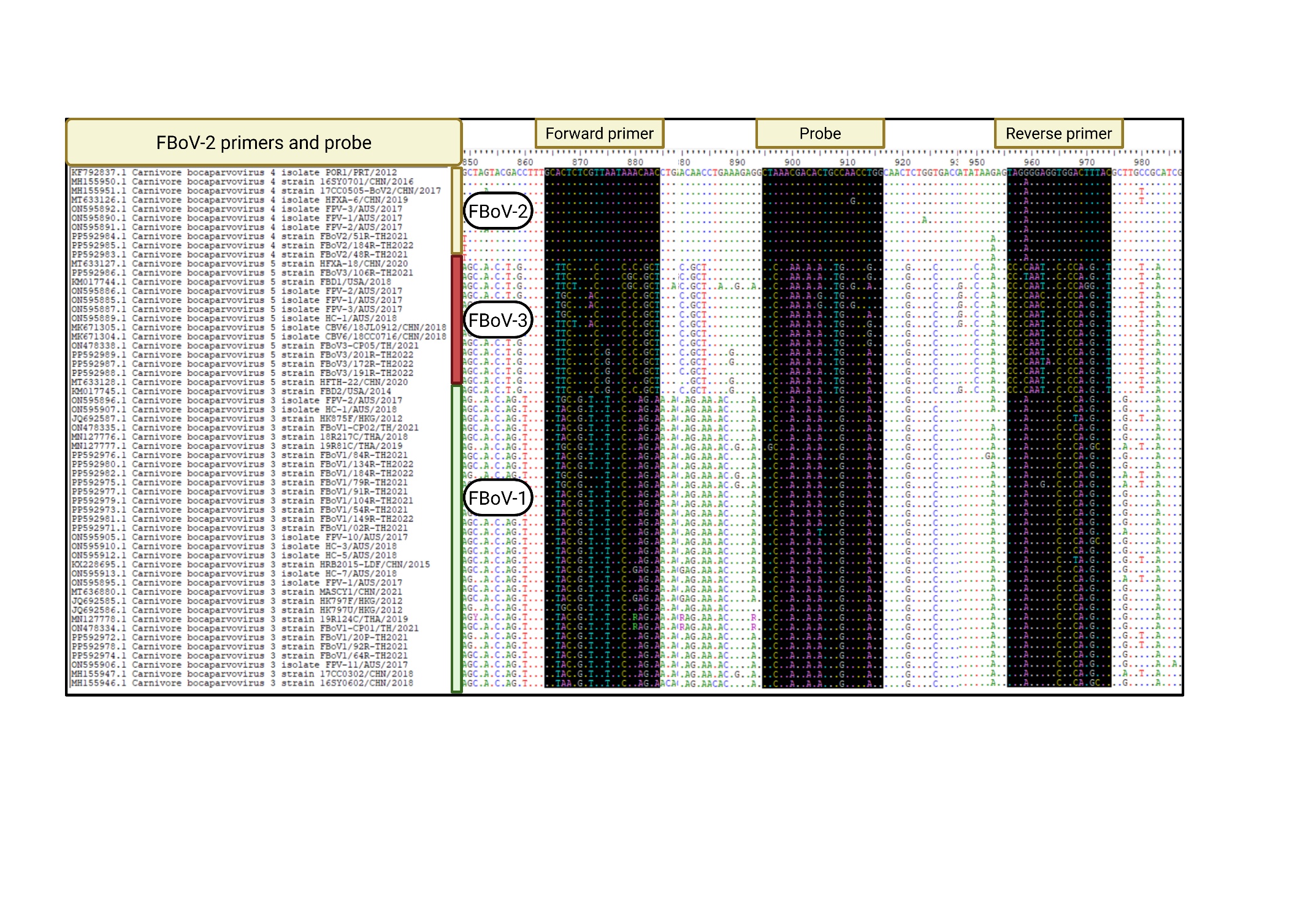
**

**Supplementary Figure S2. Alignment of the *NS1* gene sequences targeted by the multiplex qPCR assay for FBoV**-**2.** Multiple sequence alignment of the *NS1* gene region targeted by the FBoV**-**2–specific qPCR assay. All FBoV**-**2 sequences at the target region were compared with representative sequences of FBoV**-**1 and FBoV**-**3. The regions targeted by the forward primer, probe, and reverse primer are highlighted in black and enclosed within yellow rectangular boxes. The color bar on the left distinguishes the three bocaviruses: green for FBoV**-**1, yellow for FBoV**-**2, and red for FBoV**-**3.

**
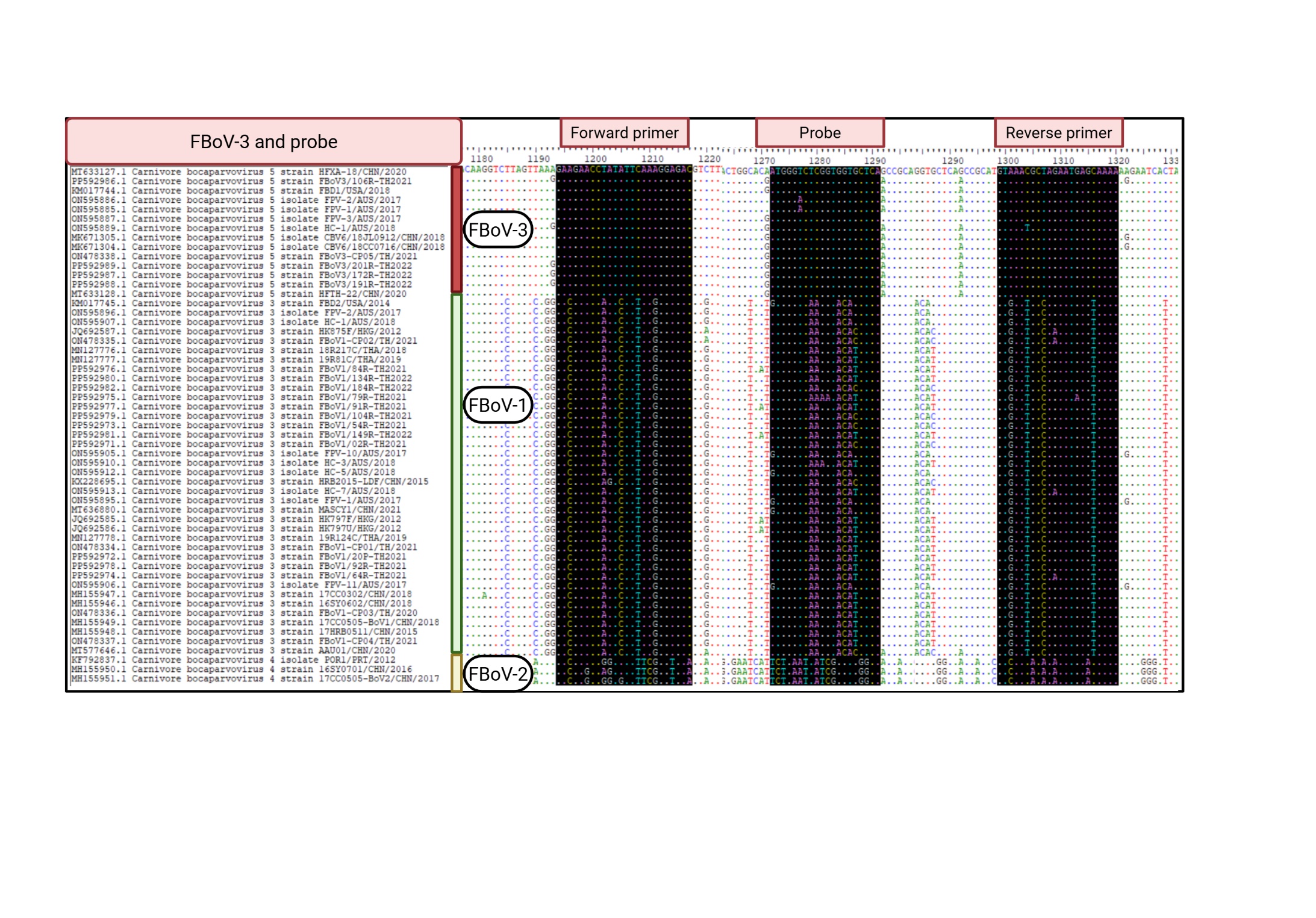
**

**Supplementary Figure S3. Alignment of the *NS1* gene sequences targeted by the multiplex qPCR assay for FBoV**-**3.** Multiple sequence alignment of the *NS1* gene region targeted by the FBoV**-**3–specific qPCR assay. All FBoV**-**3 sequences at the target region were compared with representative sequences of FBoV**-**1 and FBoV**-**2. The regions targeted by the forward primer, probe, and reverse primer are highlighted in black and enclosed within red rectangular boxes. The color bar on the left distinguishes the three bocaviruses: green for FBoV**-**1, yellow for FBoV**-**2, and red for FBoV**-**3.


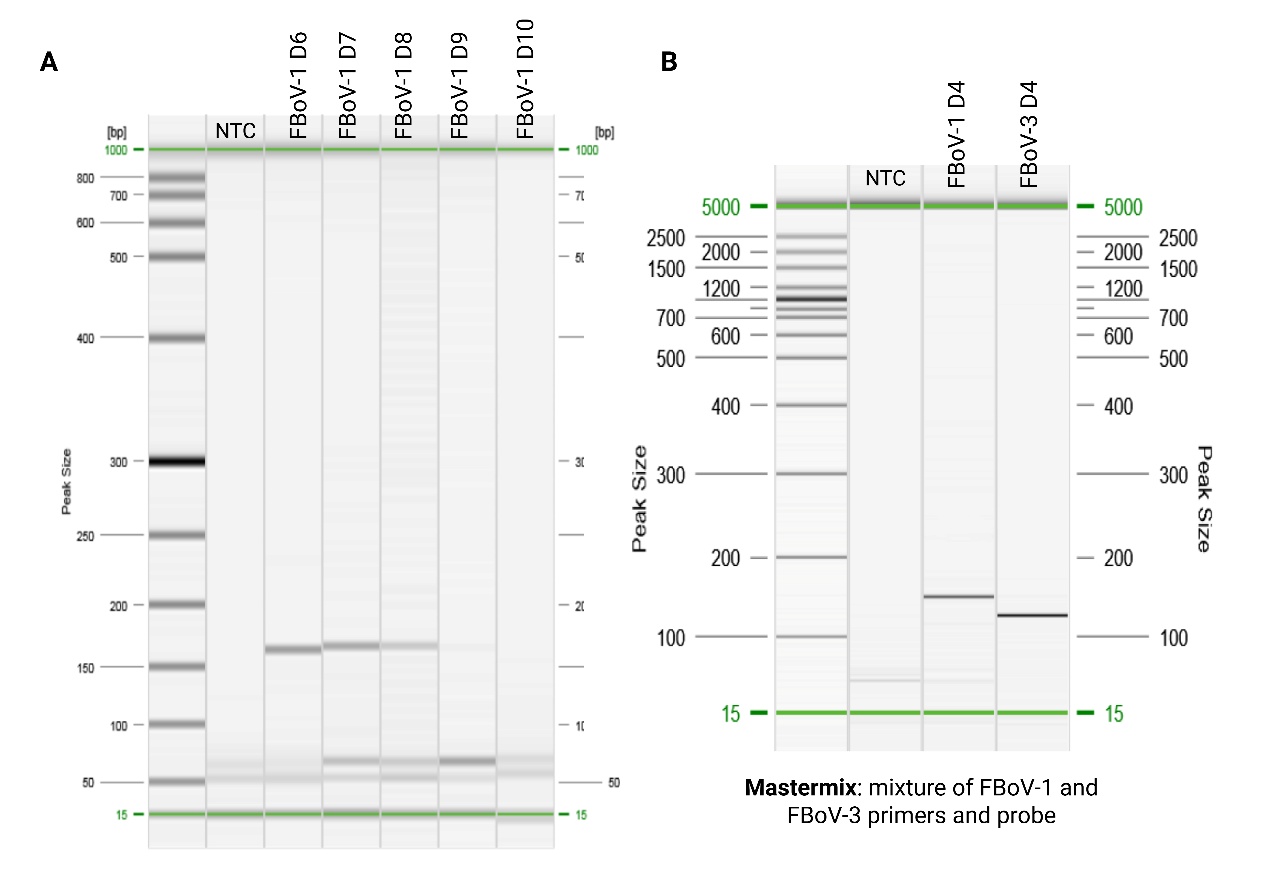


**Supplementary Figure S4. Representative capillary electrophoresis analysis during multiplex qPCR optimization. (A)** Capillary electrophoresis results of FBoV-1 dilutions showing expected target amplicons together with low-molecular-weight bands consistent with primer-dimer formation in some highly diluted plasmid standards. In the no-template control (NTC), late amplification signals were detected in the FBoV-1 fluorescence channel during qPCR analysis; however, capillary electrophoresis demonstrated only low-molecular-weight bands without the expected target amplicon band. **(B)** Capillary electrophoresis analysis of NTC reactions containing mixed FBoV-1 and FBoV-3 primer–probe sets during further assay optimization. The NTC lane showed low-molecular-weight bands consistent with primer-dimer formation in the absence of template DNA. Because late amplification signals were consistently observed only in the FBoV-1 fluorescence channel, potential interactions involving the FBoV-1 primer–probe set were further optimized. The FBoV-1 and FBoV-3 positive controls showed the expected target amplicons.
